# Developing Community Co-designed Scenario-Based Training for Police Mental Health Crisis Response: a Relational Policing Approach to De-escalation

**DOI:** 10.1007/s11896-022-09500-2

**Published:** 2022-02-27

**Authors:** Jennifer A. A. Lavoie, Natalie Alvarez, Yasmine Kandil

**Affiliations:** 1 Departments of Psychology and Criminology, Faculty of Human and Social Sciences, Wilfrid Laurier University, Brantford, ON Canada; 2 Theatre and Performance Studies, School of Performance, Ryerson University, Toronto, ON Canada; 3 Department of Theatre, University of Victoria, Victoria, BC Canada

**Keywords:** Police, Training, Mental health, Crisis, Partnership, Relational policing

## Abstract

Using the current empirical landscape of police responses to people in mental health crisis as a backdrop, this methods paper makes an argument for the central role of collaborative co-design and production by diverse community experts and stakeholders to build transformative specialized training for frontline officers. Subject matter experts (SMEs) from across key domains participated in focus groups and curriculum creation, with outputs being the co-development of a conceptual approach and an innovative experiential learning training program. Part 1 unpacks the team’s conceptual development of a *relational policing approach*. This humanized method is shaped by procedural justice, trauma-informed, person-centred, and cultural safety frameworks. Part 2 details the co-production of a novel problem-based training method for a police service in Southern Ontario, Canada. The program centres on the acquisition of core competencies related to relational policing, de-escalation, and mental health crisis response. The training was designed to bring learners through a spectrum of authentic crisis scenarios: from observer-participant scenarios informed by Forum Theatre methods and targeted SME feedback to a range of high-fidelity assessment simulations that test officers’ abilities to effectively communicate, de-escalate, and make decisions under stress. This program offers repeated opportunities for officers to practice alternative crisis management strategies in scenarios that might otherwise result in the use of force.

## Introduction

It is well documented that contact between police officers and people with mental health or substance use problems, often in crisis, is routine, recurring, and on the rise (Livingston et al. 2014; Livingston 2016; Taheri 2016). Police officers have been described as a “de facto” response to mental health crisis situations given the paucity of mental health services in the community (Baker and Pillinger 2020; Iacobucci 2014). Unfortunately, encounters between police officers and people experiencing mental illness or crisis are all too often reported to be negatively experienced (van der Meulen et al. 2021), involve a heavier reliance on the use of force (Hallett et al. 2021; Kesic et al. 2013; Moribito et al. 2017), and disproportionately end in fatality (Fuller et al. 2015; Saleh et al. 2018). Growing public dissatisfaction with police responses to mental health crisis intervention, coupled with waves of commissioned reviews and inquiries following fatal police interactions (e.g. Dubé 2016; Iacobucci 2014), have culminated in ardent calls for police reform. Among numerous recommendations to address concerns are those that centre on the development and delivery of “specialized police response” training for frontline officers aimed at enhancing mental health knowledge and prioritizing de-escalation (Pelfrey and Young 2020, p. 2).

The delivery of mental health training programs to law enforcement personnel has increased over the past 20 years (Fiske et al. 2020). Yet, effective police practice of de-escalation and crisis intervention remains elusive as evidenced by prevailing negative outcomes for people in mental health crisis (PMHC) who are in contact with the law. It has been consistently reported that police officers receive insufficient training in responding to mental health calls for service and, specifically, have training gaps in de-escalating these situations (Coleman and Cotton 2014; Dubé 2016; Iacobucci 2014). The issue of mental health crisis response is multi-faceted, far-reaching, and requires a co-operative effort to resolve – indeed, this concern is not just a “police problem”. As such, there is a clear need for enhanced involvement of community experts and stakeholders as partners in producing mental health crisis training in order to achieve a monumental shift in how the needs of people in crisis are addressed by a modern police service (Coleman and Cotton 2014; Usher and Trueman 2015).

This paper begins with a literature review on the policing of mental health crisis and focuses specifically on training issues. We then impart our methods in building community partnerships and share products derived from this deliberate collaborative processes, namely conceptual developments and a novel training paradigm. A partnership approach was achieved by assembling a multi-perspective team of researchers in the humanities and social sciences, community mental health experts, police trainers, and stakeholders such as people with lived experience of mental illness. This diverse working group co-conceptualized a unique competencies-based approach to generate transformational change for mental health crisis response. Based on these advancements, the team co-designed and delivered an innovative problem-based scenario training program that focused on relational policing approaches and safely integrating de-escalation techniques into practice. The experiential learning model was pedagogically driven by best practices in adult-based education, applied assessment, and embodied problem-based learning derived from Forum Theatre. The training model fosters applied learning through carefully designed immersive simulations featuring a diverse series of high-fidelity mental health crisis situations. Engagement in these scenarios allowed learners to rehearse competencies in the stress of the encounter and receive targeted feedback from subject matter experts (SMEs). The program is bookended by scenario-based assessment opportunities in which learners have the opportunity to demonstrate competencies acquisition by comparing performance in a series of scenarios after completing the curriculum to a baseline scenario attempted prior to undertaking the program.

## Literature Review

Reported prevalence rates of mental health-related calls range drastically from 1% to as high as 30% of calls received by police services; this variation is due in part to a lack of uniformity in how police organizations classify and report official data (Coleman and Cotton 2014; Livingston 2016; Huey et al. 2021). Moreover, there are early indications that mental health crisis calls requesting assistance from police increased during the COVID-19 pandemic (Statistics Canada 2020). It is well documented that people with mental illness have more frequent contact with police than those without a disorder (Desmarais et al. 2014a), leading to a higher incidence of arrest, particularly for minor crimes (Brink et al. 2011; Charette et al. 2014). While there are many reasons for police contact among those with mental illness, most encounters are not in response to the perpetration of crime, but instead primarily involve mental health crisis circumstances or the management of social disorder (Standing Committee on Public Safety and National Security 2014). To this point, homeless individuals living with mental illness are highly over-represented when it comes to police contact (Huey et al. 2021).

The expansion of the police service role to include mental health crisis response can be attributed to a growing population of people with mental illness living in the community where mental health services and resources are woefully fragmented, underfunded, and difficult to access (Kesic et al. 2013; Richmond and Gibbs 2020; Usher and Trueman 2015). Consequently, and often as a last resort, citizens turn to the police service for help given that these services are available around the clock. In many regions across the globe where a dedicated mental health emergency infrastructure simply does not exist, police have assumed extended responsibilities in responding to mental health crises under their mandate to preserve public safety (Baker and Pillinger 2020). As a host of community advocates and organizations have observed, one of the consequences of police serving as first responders in mental health crisis events under the mandate of “public safety” is that it criminalizes mental health crisis and perpetuates misleading and stigmatizing perceptions of individuals in mental health crisis as unpredictable and prone to violence.

### Mental Health Crisis

A mental health crisis is a situation in which a person is experiencing acute distress, disorientation, or disturbance in their thinking, emotions, or behavior that places them at risk for experiencing harm or harming others (e.g. Brister 2018). Examples of a mental health crisis include: extreme agitation, engaging in self-harm or suicide-related behavior, substance overdose, and being out of touch with reality. Crisis is distinct from mental illness, which refers to a broad range of enduring disorders that affect a person’s mood, cognition, behavior, and ability to function, such as depression, bipolar disorder, anxiety disorders, psychotic disorders, and post-traumatic stress disorder (Mayo Clinic 2021). While crises often occur among people with mental illness, mental illness is not a necessary precipitating condition—*any* individual can experience a mental health crisis. Service providers, family members, employers, bystanders, or the person in crisis themselves are the most likely people to summon the police for help during a crisis (Shore and Lavoie 2019). Mental health crises can vary widely in presentation and seriousness. Accordingly, police are dispatched to respond to PMHC through a variety of calls overtly related to mental health issues, commonly: “mental-health call”, “suicide risk”, “wellness check”, “missing person”, “absconding”, and “intoxicated person”. Police are also dispatched to a wide range of criminal offence calls, for example, domestic disturbances, where there can be a mental health crisis component underlying the call. Consequently, officers may not know they are responding to a mental health crisis based on dispatch information until they arrive and engage those on scene.

### Policing Practice, Training Styles, and Compliance

In practice, responding to PMHC can be the most challenging and uncertain of calls that police constables confront in the field (Pelfrey and Young 2020). Police arrival and presence alone can be escalating for PMHC who are typically fearful, often of authority figures. In many instances, PMHC do not respond well to standard policing tactics that tend to be more effective in gaining swift compliance with a member of the public in the context of law enforcement (e.g. shouting verbal commands, using intimidation or physical force to gain compliance; Baker and Pillinger 2020; de Tribolet-Hardy et al. 2015). Authoritarian tactics and forceful styles often have an aggravating effect on situations involving a mental health crisis, and ultimately increase the risk of injury to a distressed person or officer (Kesic et al. 2013; Kleining 2014; Watson et al. 2008b). Complicating this, “stress-based training”, which emphasizes vigilance and military-like preparation to threats in the field using defensive tactics, force and weaponry, cultivates a “warrior” mindset. Such an orientation may prime officers to view non-compliant or dysregulated behaviors as more perilous than they are, and resort unnecessarily to the use of force (Li et al. 2021).

Those in mental distress are typically less likely to comply with initial police directives (Kesic et al. 2013), owing to challenges in information processing and communication due to conditions such as anxiety, psychosis, or paranoia. A person in crisis may present as if they are ignoring police instructions, when in reality they are unable to listen or respond in a timely way (de Tribolet-Hardy et al. 2015). Officers report that they often perceive PMHC as non-compliant and resistant (Kesic et al. 2013), and correspondingly are more likely to use more coercive tactics to gain compliance in these situations (Fyfe 2000; Hallett et al. 2021). Sometimes, officers use force to subdue individuals perceived as aggressive or erratic and do not recognize that the person is behaving in this manner due to a mental health crisis (Godfredson et al. 2011). Indeed, there is evidence that police officers fail to detect a significant proportion of mental health and physical morbidities among detainees, and have trouble identifying individuals considered to be “vulnerable” (McKinnon and Grubin 2013). Officers must be able to recognize cues of mental health crisis in order to promote safety and respond in ways that are most helpful to the citizen (Oliva et al. 2010; Richmond and Gibbs 2020). For example, the behavior of a person in crisis is often shaped by an officer’s own approach and actions (e.g. body language, communication style, time investment; Canada et al. 2012; Pollack and Humphries 2020). Thus, constables who are able to accurately recognize mental health crises can make use of specialized techniques, including de-escalation where possible, to foster safety and avoid unnecessary escalations.

### Misperceptions of Dangerousness and Impacts of Stigma

In situations where officers do recognize signs of a mental health problem, they may act based on stereotypical misperceptions of people with mental illness as inherently dangerous, violent, unpredictable, and unable to be reasoned with (Ruiz and Miller 2004; Wittmann, et al. 2020), amplifying the odds of using force (Rossler and Terril 2017). Prejudice towards those with mental disorders can take the shape of fear, anger, and pity (Corrigan 2005). Indeed, a significant proportion of officers reports fear of injury (Wittman et al. 2020), worry, uneasiness, and feeling threatened when interacting with individuals with mental illness (Ruiz and Miller 2004). Uncertainty related to responding to mental health crisis calls contributes to a heightened perceived risk of danger, increasing the likelihood of police use of force (Fyfe 2000). Unlike mental health professionals who know their clients and provide services in a familiar setting, police officers often respond to unknown persons in ambiguous circumstances in unfamiliar environments where risks associated with the location are unknown (de Tribolet-Hardy et al. 2015). They arrive on scene with little prior background knowledge about the individual, their mental state, catalyst for the crisis, or safety risks. Officers who perceive a threat of risk are more likely to use force to manage the situation (Richardson et al. 2019). The ambiguous setting that typifies an officer’s daily work underscores reasons why addressing bias is essential to promoting safety and reducing unnecessary force.

Opposite to commonly held stereotypes, research shows that serious mental illness on its own accounts for little community violence (Swanson et al. 2015). This risk for violence tends to be related to specific symptoms that cause a person to feel threatened (e.g. paranoia) or that their personal control is being overridden (e.g. command hallucinations; CMHA Durham n.d.). People with mental illness are no more likely to injure police than those without a disorder (Kesic et al. 2013). Those with mental illness are more likely to be victimized rather than engage in violence (Burczcka 2018; Desmarais et al. 2014b). A far superior predictor of violence is co-occurring substance use (Van Dorn et al. 2012; Swanson et al. 2015). Intoxication may serve as a trigger to mental health crisis incidents (Short et al. 2013). Thus, an officers’ ability to accurately assess risk for violence, particularly in mental health crisis situations, is vital and can be improved when biased attitudes are identified and addressed. Ultimately, stigma directed towards citizens with mental health concerns contributes to perceptions of these community members as devalued, and can negatively impact an officer’s willingness to safeguard the person’s wellbeing or provide an equitable level of service,

### Disproportionate Use of Force

While the use of lethal force by police is exceptionally rare, international policing research literature has consistently shown that people with histories of mental illness are disproportionately killed in police interactions (Fuller et al. 2015; Nicholson and Marcoux 2018; Saleh et al. 2018). The International Association of Chiefs of Police defines “force” as “that amount of effort required by police to compel compliance from an unwilling subject” (IACP 2001, p. 1). While the majority of police interactions with the public are resolved without force, the use of force is aimed far more commonly toward people living with mental illness compared to those who are not (Kane et al. 2018; Moribito et al. 2017). One study found that cases in which force was used commonly featured a person who was intoxicated, behaving aggressively, possessed a weapon, and had a diagnosis of psychosis or schizophrenia (Kesic et al. 2013). An integrative review on conducted energy weapon use found that tasers were more likely to be deployed on people experiencing mental distress, particularly those with comorbid substance use issues, as compared to cases of criminal arrest, and that those in crisis were subject to longer lasting and a greater number of taser shocks (Hallett et al. 2021).

### Consumer and Family Perceptions

Research reveals that people with lived experience of mental illness perceive police contact in a mixed light. Desmarais et al. (2014a) found that while consumers held generally positive perceptions of the police, compared to the general population they rated police less favorably with respect to enforcing laws, approachability, providing information, and treating people fairly. Livingston et al. (2014) study of those with mental illness found that the majority reported being treated fairly in recent police interactions; however, most also reported enduring some form of police force in the past, with a third deeming this force excessive. Wittmann et al. (2020) found that police encounters were chiefly experienced as positive, respectful, and non-threatening. Other recent research indicates that people with mental illness view police experiences as decidedly negative (Goldberg et al. 2019; van der Meulen et al. 2021), which can hinder future help-seeking, trust in police, and perceptions of police legitimacy. Relatedly, families report fear in calling police for assistance during a loved one’s mental health crisis and cite concerns about excessive use of force and lack of police training in responding to mental health crisis (Baker and Pillinger 2020; Lavoie 2018).

### Specialized Police Training

To address rising contacts with PMHC, many police services have, among other resolutions such as launching co-response units with mental health professionals (Cotton and Coleman 2017; Pollack and Humphries 2020), focused on “specialized policing response initiatives” to train frontline officers to recognize mental health problems, de-escalate crisis, and divert people to treatment and supports (Pelfrey & Young, 2020, p. 2). Police officers are usually the first and sole emergency responder to mental health emergencies (Richmond and Gibbs 2020), yet generally, they have been inadequately trained to manage mental health crisis interactions (Coleman and Cotton 2014; Dubé 2016; Iacobucci 2014). Officers report feeling ill-equipped to respond to mental health calls (Wells and Schafer 2006). Mental health training has been increasingly offered to police services over the past two decades (Fiske et al. 2020). One of the most widely implemented specialized response training programs is Crisis Intervention Team (CIT) Training. CIT was designed to foster safer encounters between police and those in mental health crisis with an emphasis on de-escalation techniques, mental health knowledge, and diverting citizens towards mental health services (Canada et al. 2012; Pelfrey and Young 2020). While CIT has been shown to positively impact knowledge and attitudes about mental illness and enhance referral to services, a research review found that the program showed no significant effects on reducing arrests or dampening the use of force against people with mental illness (Taheri 2016). There are a myriad other police training programs that have been aimed at improving police responses to escalated situations involving people in mental health crisis. We are not, however, aware of any examples that originated using a grassroots co-design and co-production approach.

### Recommendations and Partnerships

Given that most mental health education is delivered only in lecture format, Fiske et al. (2020) recommend increased incorporation of skills-based learning that offers the opportunity to practice skills. Applied modalities such as role playing and interactive scenario-based training provides experiential learning opportunities to rehearse de-escalation and communication strategies. In his 2016 report, Ontario Ombudsman Paul Dubé identified the comparatively diminished attention given to de-escalation and communication techniques in the training of police constables. He further noted that “a final pass/fail test on the use of force is largely about using force, not using judgement to de-escalate” (Dubé 2016, p. 7). Training has been left to the discretion of individual police organizations with no continuity from service to service and no government oversight (Dubé 2016, p. 7). As Coleman and Cotton’s (2014) expansive examination of training programs in a host of academies and divisions across Canada reveals, scenario training is widely used but its efficacy is taken as a given in the absence of partnerships between police training programs and university researchers who can assist in undertaking such a dedicated research evaluation. A key recommendation urges police services to “collaborate with researchers […] to develop a system for collecting and analyzing standardized data regarding the effectiveness of training” (Iacobucci 2014, p. 155). There are robust calls for mental health crisis education to include people with lived experience of mental illness themselves as well as family members, clinicians, mental health nurses and advocacy associations (Coleman and Cotton 2014; Iacobucci 2014; Usher and Trueman 2015). Taken together, transformative training advancements may be achieved through the gathering and collaboration of key community stakeholders involved in mental health crisis response, and building programs with the intention of evaluation at the forefront of design.

### Present Study and Objectives

The current study profiles the collaborative work resulting from an intentional partnership approach realized by gathering a multi-perspective team of researchers in the humanities and social sciences, community experts and stakeholders including clinicians, nurses, crisis workers, people living with mental illness, addictions experts, cultural safety specialists, advocacy association heads, as well as police trainers from numerous rural and urban jurisdictions in Southern Ontario, Canada. The project was guided by three objectives designed to transform police training in de-escalation and crisis intervention:

1. To engage people living with mental illness as well as community stakeholders including mental health professionals and organizations to foster partnerships with law enforcement;
2. To co-conceptualize optimal competencies in police response to mental health crisis;
3. To co-design and co-develop a problem-based scenario training curriculum and assessment that cultivates these competencies using an innovative pedagogical method that emphasizes integrated SME feedback.

## Method and Results

### Part 1: Stakeholder Partnership Building and Co-conceptualization

There is widespread consensus that scenarios dedicated to mental health crisis education need to be amplified and, most critically, involve people with lived experience of mental illness as well as clinicians and advocacy associations (Coleman and Cotton 2014; Iacobucci 2014). One of the key ways of reducing stigmatizing attitudes toward PMHC is not only to increase mental health crisis education (Compton et al. 2014; Pinfold et al. 2003), but also to increase the opportunities for positive social engagements between police and those with lived experience of mental illness (Dalky 2012; Thornicroft et al. 2008). Involving people with lived experience in the design, delivery, and assessment of the training program was pivotal to the realization of our project’s core objectives in reducing stigma and improving the overall quality of interactions between police and PMHC. Moreover, scenario training is rarely led by individuals with experience in problem-based scenario training as an educational method within adult learning contexts (Coleman and Cotton 2014). Without third-party facilitators with experience in problem-based scenario training and adult learning who can guide the creation and development of scenarios from all perspectives—police, people with lived experience of mental illness, community advocates, and clinicians included—scenarios risk offering only one-sided perspectives; they will remain, in other words, scenarios created “by officers for officers” with the potential to privilege the exigencies of legal concerns and governing mental health acts at the expense of procedural justice. The development of mental health crisis training requires a fundamentally transdisciplinary approach since the complexity of mental health crisis response exceeds the capacity of any one disciplinary arena of expertise. Given the stakes of community safety at play, mental health crisis training also depends on a meaningful coproduction process between researchers and community partners, which centres on consumers who determine what adequate response and care looks and feels like for them.

Taking as its impetus the key recommendations that emerged from “TEMPO: Training and Education About Mental Health for Police Organizations” (Coleman and Cotton 2014), our study design was driven by a coproduction process that relied on community stakeholders and those living with mental illness as the primary consumers. Aligned with the training of team members in performance, where coproduction is a common form of practice-based research and collaborative devising, coproduction depends on mutually respectful relationships where each contributing member is seen as having an integral role to play in the project. Coproduction describes “a relationship where professionals and citizens share power to deliver support together, recognizing that both partners have vital contributions to make in order to improve quality of life for people and communities” (Slay and Stephens 2013, p. 3). For the purposes of this study, coproduction telescoped on the participation of consumers, fostering respectful, collaborative relationships among those working in mental health care services and those with experience as service users (Bland and Epstein 2008). Coproduction is a vital pathway for the transformation of health care service and while this is a central message in international policy documents, there is a dearth of coproduction models within clinical mental health services (Kidd et al. 2015). As a method that brings together a diversity of stakeholders, perspectives, and investments in mental health service delivery, coproduction centralizes human rights and the value of lived experience (Kidd et al. 2015; National Consumer and Carer Forum of Australia 2004).

### Partnership Building Process

The composition of our team was determined by identifying the constellation of perspectives necessitated by the exigencies of policing and mental health crisis response that could be brought to bear on the coproduction process. The lead researchers came together on the basis of a productive cross-pollination of expertise in criminology, forensic psychology, policing and mental health crisis response; immersive, scenario-based training and cross-cultural encounters; and applied theatre and simulation in medical training contexts. Through literature reviews, the researchers identified leading thinkers in the fields of de-escalation training and mental health recovery who would be well positioned to join the research team. Local community care facilities and associations such as Ontario Shores Centre for Mental Health Sciences, the Centre for Addiction and Mental Health, and the Schizophrenia Society of Canada assisted with the recruitment of individuals with lived experience of mental illness, peer support workers, and community advocates who were interested in joining the study. The lead researchers also reached out to the directorship of San’yas Indigenous Cultural Safety in Ontario and British Columbia with an invitation to join the project. Representation of police organizations included police college instructors specializing in de-escalation training as well as mental health. Each team member joined the project after a series of conversations and meetings spanning six months, which allowed shared values, commitments, and core objectives to be identified. The time taken for these conversations allowed a working relationship of trust and mutual respect to develop, which provided a strong foundation for the project. It also allowed time to clarify the roles of each team member and the contributions they stood to make so that collaborators could proceed with a recognition of why they were vitally needed in the project and the importance of their presence in the room. A national team of 12 collaborators was finally assembled to commence the coproduction process, which also involved a core ensemble of eight actors who were integral to scenario testing and development over the course of 1.5 years. This research initiative was reviewed and approved by the REBs of three universities and one hospital affiliated with the project.

### Focus Group Procedure

In a series of eight-hour workshops over three days, the research team—including community stakeholders and people with lived experience—held in-person focus groups in Toronto, Ontario. The goal of these focus groups was to engage in program logic modeling by applying the guidelines of TEMPO (Coleman and Cotton 2014) as our planning framework to arrive at training competencies. Over a series of small group breakout sessions, in which team members shared lived, clinical, and road experience on optimal mental health crisis responses, core competencies began to emerge. Ideas were brought back to the larger group for discussion to identify thematic clusters and overlaps. The research leads then cross-checked the core competencies with extant literature on best practices in crisis intervention from the field of psychiatry and examined the list for areas of omission. The competencies working list was then matched against the provincial basic constable training program to ensure it was harmonious and worked within any existing constraints. Competencies were synthesized and further refined by the working group over the period of 1 year.

### Conceptual Development

A result of the focus group working sessions was the co-development of the concept of a *relational policing approach*, which encapsulated the identified competencies as well as learning objectives for the training curriculum developed in Part 2 of this project. Relational policing involves offering a genuine and personalized response, conveying empathy and concern for wellbeing, and taking the necessary time to cultivate a connection and build trust while managing safety risks. Indeed, it is recognized that this approach is to be used when the officer has assessed the risk and judges that it is reasonably safe to engage. As such, this approach is expected to be integrated with officer safety tactics including positive containment, the use of barriers, as well as time and distance that can increase opportunities for effective communication. To engage “relationally”, the officer brackets their authoritative positioning, conscious that they are co-implicated in the encounter and drawn into a sense of responsibility for the person’s welfare and wellbeing with the knowledge that no one (including themselves) is invulnerable to mental health crisis. Relational policing is predicated on collaborative, transparent decision-making and a reciprocal investment in relationship building, and peaceful resolution. Relationship building allows the officer to gain an informed understanding of the situation and the person’s needs to guide effective options for resolution. A hallmark of this approach is encouraging the person to share their perspective and collaborating on ways to resolve the situation together. A relational approach is a fair and non-judgmental one that emphasizes active listening and valuing the person’s point of view to better understand the situation.

**Fig. 1:**
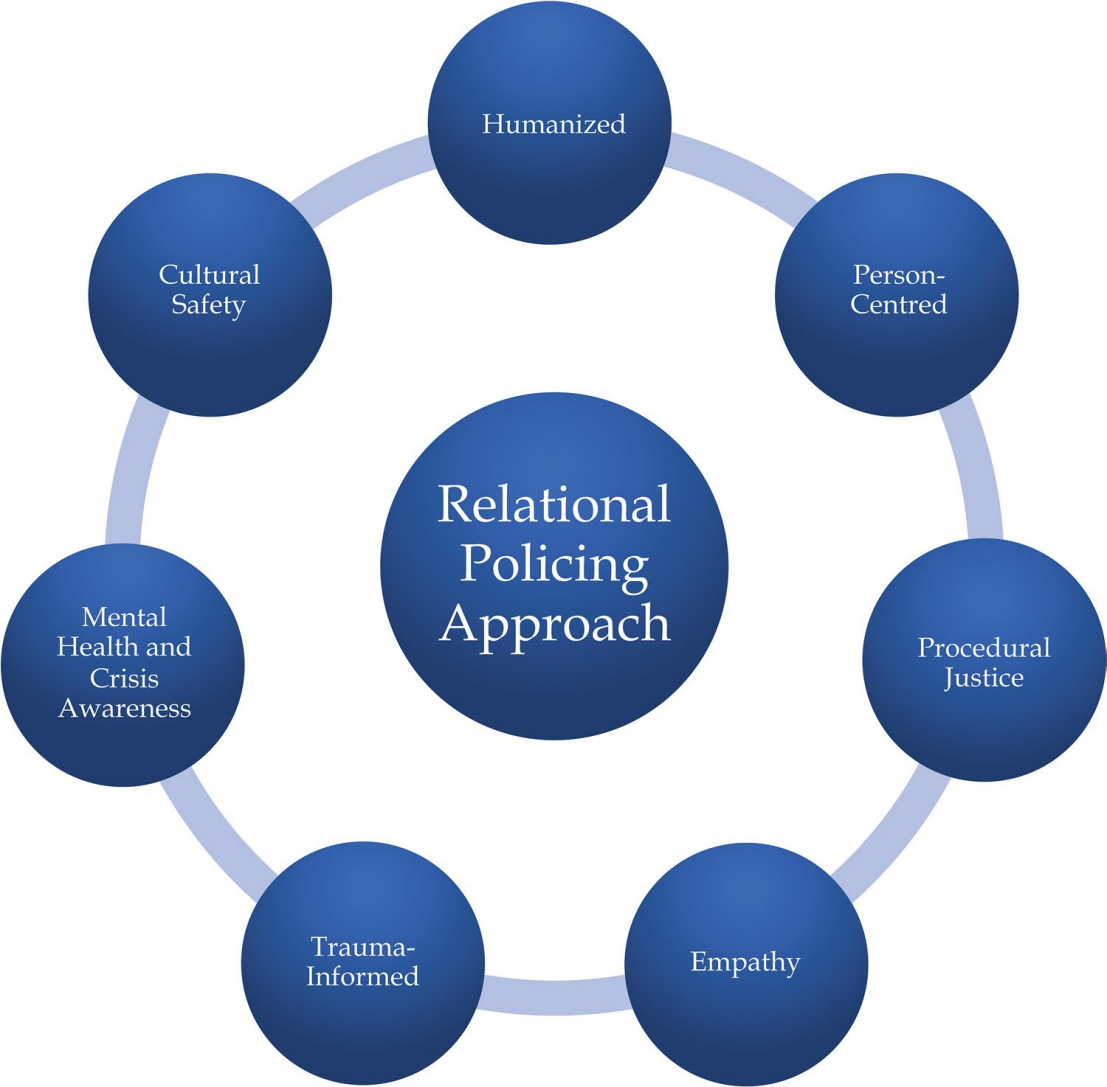
Relational policing approach

To be relational, officers must be informed about mental illness and trauma and recognize how these conditions and experiences impact a person’s ability to interact. Officers do not have to be experts in mental health to help a person in crisis. With a basic understanding of mental illness, trauma, and crisis, officers are better able to communicate with community members and help connect them to professionals while keeping them safe. A peaceful resolution to the crisis is more likely when a person feels heard, valued, and part of the decision-making process. Theoretically, being relational fosters positive encounters, increases trust and cooperation, and promotes safety and de-escalation of tense situations. Relational policing is multifaceted in its approach and includes humanized, person-centred, procedurally just, empathetic, informed, and culturally safe modes of engagement (See Fig. 1).

### Humanized

Using an authentic and humanized style to engage a PMHC is a method to connect, build rapport and trust, and reduce the fear that the person may be experiencing. Strategies to personalize the encounter, such as the officer introducing themselves by their first names, and seeking common ground with the person in crisis is key to this approach. Given that authoritative or coercive approaches are generally inadequate in helping PMHC and tend to escalate the situation further (Baker and Pillinger 2020; de-Tribolet-Hardy et al. 2015), a firm but friendly, caring, and genuine approach is likely to engender more dignity, receptivity, and cooperation. Self-awareness of what the officer is bringing to the situation is valuable (e.g. the arrival of one or many authority figure[s] can be distressing for those who conclude that they are in trouble or have paranoia specific to police). A humanistic approach includes minimizing negative effects of officer presence that do not serve the situation, such as reducing the formality of the encounter. Opting for a less authoritative approach where possible to resolve mental health crisis situations may enhance community trust in police.

### Person-Centred

Citizens expect public services to be delivered with a focus on the person or consumer (Coleman and Cotton 2014). People with mental illness and their family carers specifically expect that police officers have knowledge about the basics of mental illness, how it affects a person’s cognition and behaviour, and know how to safely resolve these calls (Brink et al. 2011; Lavoie 2018). A person-centered approach is about treating people as they want to be treated, and responding with knowledge and respect for their self-identified concerns, values, perspectives, and priorities. Central to this approach is that the person is seen as the “expert in their own mental health”, that is, they know themselves and what works best for them (Portico Network n.d.).

Police traditionally approach public encounters with a mindset that they must resolve situations quickly so that they can attend and clear calls logged before the end of shift (de Tribolet-Hardy et al. 2015). Taking the time to ask about and cultivate a fuller understanding of the situation from the person’s point of view, and taking their perspective seriously is the essence of a person-centered approach. Police personnel can collaborate with PMHC to identify concerns, assess needs, and encourage them to be actively involved in decision-making on how to resolve the situation. Actively listening to their values, needs, and concerns and effectively addressing them will help to build trust and inform the officer of potential next steps (Jones et al. 2019). For example, asking what has helped in the past when the person was feeling this way may provide alternative ideas towards resolution.

### Procedurally Just

Procedural justice focuses on the way police interact with the public, and how those interactions shape the public’s views of the police and their willingness to obey the law. Procedural justice refers to whether citizens feel that they were treated with dignity and fairness by police (Lind and Tyler 1988). People with mental illness tend to feel less distressed when encountering police if they feel they are treated fairly and their welfare is kept in mind (Watson et al. 2010). Fundamental aspects of a procedural justice framework include (Lind and Tyler 1988; Watson et al. 2010): 1) participation, which involves the PMHC being encouraged to express their point of view, feeling heard, and having the opportunity to collaboratively take part decision-making; 2) dignity, which includes being treated with courtesy and respect as valued members of the community—respect here refers to the officer acknowledging and having due regard for the person’s feelings, wishes, worth, rights, and traditions (Jones et al. 2019); and 3) trust, which includes officers showing sincere concern for a person’s welfare and citizen’s trusting police as dependable and legitimate agents of the law. 

Multiple studies show that people who felt police treated them with fairness, respect, and dignity were more likely to: cooperate with police and were more willing to work with police in the future; viewed the encounter more positively, even if the outcome involved apprehension or arrest; report greater levels of satisfaction with police services; and have more trust in police (Lind and Tyler 1988; Watson et al. 2008a). When police use their authority in a procedurally just manner*,* people feel that they are respected and valued members of the community, and the result is cooperation. Treating people fairly is vital to establishing relationships and forming a better understanding of the situation by all those involved (Coleman and Cotton 2014). On the other hand, disrespectful police responses reduce the chances of public cooperation (Watson et al. 2010). Further, extremely negative or violent encounters with police can create profound and long-lasting negative impressions of police that cannot be repaired by past or future positive experiences with police (Coleman and Cotton 2014).

### Empathetic and Trauma-Informed

Many people with whom officers routinely interact are affected by life-altering, traumatic experiences that create turmoil. It is estimated that ¾ of the population has experienced at least one traumatic event in their lifetime (Klinic 2013). The impacts of trauma are far reaching and can contribute to mental illness, substance use, suicide, chronic illness, and other negative outcomes (Alberta Health Services 2019). Certain groups of people are at greater risk for experiencing trauma, such as children with histories of abuse, Indigenous peoples, homeless people, refugees, people with serious illness, military personnel, and first responders (Centre for Suicide Prevention n.d.)

A trauma-informed approach involves an intentional effort to take into account the impact that traumatic experiences have had on a person. Officers do not have to be trauma experts to employ this method. This approach requires a shift in perspective that moves away from an assumption of fault toward an approach that considers a person’s circumstances and history (Rosenberg 2011). A trauma-informed approach focuses on the way officers *relate* to people using empathy and compassion. Empathy is the ability to comprehend another person’s situation or feelings as well as share another person’s feelings. Knowing the signs of trauma, how it affects people, and recognizing that individuals who are traumatized may behave out of fear can cue officers to provide both physical and emotional safety (e.g. reassurance, offering choice, providing structure to the situation).

### Mental Health and Crisis Awareness

Having the capacity to inquire about and identify basic signs and symptoms of mental illness and mental health crisis is essential given that these conditions can be missed by responding officers (Godfredson et al. 2011; McKinnon and Grubin 2013). Discerning cues can assist the officer in adapting their engagement to foster effective communication and intervention (e.g., modifying pace and paralanguage when communicating with a person with auditory hallucinations) and help officers feel more confident when arriving at crisis calls. Distinguishing accurately between signs of mental illness versus intoxication is of particular importance. Employing specialized strategies such as removing distractions, using simple language, and taking care that officers speak one at a time will enhance communication. Appreciating that those in crisis may require additional time and patience to respond to police instructions may reduce officer frustration or misinterpretation of a person’s response as deliberate non-compliance.

The most damaging stereotype directed towards people with mental illness is that they are inherently dangerous. Encouraging self-awareness and addressing stigmatizing attitudes among officers directed towards mental illness is vital to relational policing. Officers should possess an accurate understanding of violence risk cues and strive to ascertain intentions to avoid misinterpretation of behavior. The inclusion of people with mental illness in police mental health training has been recommended (Coleman and Cotton 2014) to bolster understanding and compassion for lived experience and reduce bias.

### Cultural Safety and Intersectionality

A final feature of relational policing emphasizes skills in self-reflection as a way of promoting a respectful and safe encounter with equity-deserving communities such as Indigenous peoples, racialized populations, LGBTQ2 + , and people with disabilities who experience multiple and intersecting forms of inequality, bias, and discrimination (Krenshaw 1989). While cultural safety is a concept that has advanced primarily in healthcare delivery contexts, our collaborators specializing in Indigenous cultural safety have demonstrated that its core principles have a wide range of applicability to other arenas of practice including government policy and service (Brascoupe and Waters 2009). When applied to the context of policing and mental health crisis response, cultural safety encourages officers to be self-reflexive about power differentials, that is, about the authority they hold in the encounter and the impact of their presence on the person in mental health crisis. This self-reflection includes a consideration of how social and historical contexts, such as the ongoing impacts of colonization and the structural imbalances it has produced, as well as biases, prejudices, and racism, shape perceptions and interpersonal encounters (Indigenous Physicians Association of Canada 2008).

Cultural safety is a relatively new concept that presents a paradigm shift away from the impasses produced by more common approaches to cultural difference such as “cultural competency”, which promotes a way of thinking about culture as a fixed, static phenomenon that one can know or understand, reducing culture to a set of skills one can master (Baba 2013). It also avoids the potential inadequacies of “cultural sensitivity”, which focuses attention on the “other” as a bearer of culture rather than individual self-reflection, reinforcing the position of the dominant culture and leaving power imbalances intact. A culturally safe approach recognizes that one can never be truly competent in a culture other than one’s own. Importantly, cultural safety as an approach dovetail with the principles of relational policing, person-centred response, and procedural justice in that persons in mental health crisis determine what “safe service” means and looks like for them: the needs and voice of the person in crisis take a predominant role (Indigenous Physicians Association of Canada 2008). The integration of cultural safety in de-escalation strategies remains an imperative in light of recent investigations revealing that Black, Indigenous, and racialized populations in mental health crisis are subject to disproportionate levels of violence by police (Nicholson and Marcoux 2018).

### Part 2: Co-development and Co-delivery of Scenario-Based Curriculum

In a series of workshops, the research team broke into several pods comprised of three to four team members representing different disciplinary perspectives, lived experiences, and practices. Each pod was invited to share stories of an officer and a person in mental health crisis, which they had either witnessed or experienced first-hand. Pod members discussed the nature of the mental health crisis, the dynamics of the encounter, the behaviours and decision-making of the officers, how or whether the crisis was resolved, and the lasting imprint that encounter had left on them and why. The pod was then tasked with the development of an outline for a live action scenario, which could be adapted from one or more of the stories shared. To guide their scenario development, the pods were given a series of questions that asked its members to consider the demographics of the person in crisis; the location, context and precipitating circumstances that brought the police service to that site; the history and backstory of the person in crisis that have informed this moment; whether other people are present and, if so, how they are reacting to the PMHC and the officers; and finally, what they expect the trainee would do to engage effectively with the PMHC. The pods shared their scenario outlines and offered insights on optimal trainee response. Team members underscored the importance for the trainee to learn the backstory of the PMHC as a way of humanizing the encounter, reducing stigmatizing attitudes and interrupting stereotypes, orienting an approach that asks not *“What is wrong with you?”* but rather *“What has happened to you?”* (Rosenberg 2011).

The project leads collected and reviewed the scenario outlines produced by the pods, analyzing the content for commonalities in theme, given circumstances, the scope of mental health crises and diversity of demographics represented. Alongside these scenario outlines, the project leads examined coroner’s inquest reports in Canada dating back to 2011 involving police use of lethal force in response to individuals in mental health crisis. The research team chose a total of eight scenario prototypes for concentrated development on the basis of the diversity of individuals and contexts represented. The team also made the selection by considering the range of learning opportunities they collectively offered with respect to the scope of mental health crises at play and the degrees of imminence requiring intervention and assistance. Drafts of these scenario outlines were circulated among team members for feedback from all perspectives and collectively edited to ensure authenticity in the description of PMHC backstory, mental health crisis signs and symptoms, and optimal officer response.

Over a period of 1 year, the research team conducted a live testing process with each of the scenarios. The project leads worked with professional actors and police officers who engaged in a series of improvisations over several days, allowing the research team to examine variations on the encounter depending on the scope of mental health crisis behaviours and officer decision-making. In turn, this iterative rehearsal process gave actors extensive opportunities to improvise in response to possible courses of action while guiding the officer toward key learning objectives, reinforcing the officer with increased communication and decreased agitation when the officer effectively demonstrated core de-escalation competencies. A provisional version of each scenario was presented to the full research team in the form of an “open rehearsal” process in which team members were invited to vet the scenario and offer feedback. Once the research team arrived at a consensus that the scenario had achieved a satisfying level of fidelity, scalable challenge levels for a range of learners, and optimal opportunities for competency-based learning, the research leads engaged in a final series of rehearsals that concretized, polished, and refined the scenarios in preparation for training program delivery. Before commencing the training with learners, the team spent over 200 h developing a total of eight scenarios. The team moved into a final phase of scenario refinement once sets pieces were in place to establish the context of the encounter as well as the parameters of the environment and enhance the immersive effects of the simulation.

### The Forum Theatre Method and Scaffolded Rehearsal Learning

The study’s coproduction process advanced a form of problem-based scenario training informed by Forum Theatre (FT) methods adapted from the Brazilian director Augusto Boal’s (1979) methodology. Based on the work of Brazilian educator Paulo Freire’s *Pedagogy of the Oppressed* (1970), Boal’s Forum Theatre methods are anchored in a philosophy of “transitive, two-way learning and collective empowerment” (Cohen-Cruz 2010, p. 43), which engages learners in techniques of practice-based learning. As a form of embodied learning, Forum Scenarios are particularly effective in adult learning and professional education contexts as a way to foster knowledge synthesis and long-term retention (Beckett and Morris 2001; Beaudoin 1999; Chapman 1998) as well as “reflection-in-action”: a method of learning in practice that advances adult learners’ capacity to “think on their feet” and immediately change direction or course of action (Merriam and Bierema 2014, p. 116). Our Forum Scenarios were designed to foster the agility that comes with reflection-in-action by focusing attention on how learners transition between de-escalation techniques in response to the fluidity of the encounter—a skill set that is especially critical in person-centred and relational policing.

In practice, the Forum Scenario was a form of problem-based, observer-participant scene work whereby the trainees moved from being observers of a staged scene to participants, engaging in stop, rewind, and playback techniques that provided repeated opportunities to test approaches and try alternative crisis resolution strategies. A team of four SME instructors, comprised of a mental health clinician, a person with lived experience of mental illness, community mental health advocate, and de-escalation trainer, offered feedback and shared observations at critical moments of the scene work that were “paused” in action in order to demonstrate in immediate terms *how* information—that would otherwise be delivered in a de-contextualized lecture format—was directly applicable and pivotal to ethical decision-making and effective communication in the encounter with PMHC. In this respect, the Forum Scenarios fostered a multi-directional flow of knowledge and experience to engage trainees in collaborative problem-solving. The pause, rewind, and playback techniques of the Forum Scenario harnessed key “teachable moments” allowing the learner to receive constructive feedback from instructors at pivotal moments so that best practices could be rehearsed and immediately integrated into the learner’s repertoire.

To enhance long-term retention of de-escalation, communication, and relational strategies with PMHC, as well as an ability to apply these strategies under stress, our project offered a series of scenarios that unfolded in several stages along a spectrum: from the controlled method of stop-playback, Forum Scenario work to an intensive circuit of assessment scenarios (“Circuit Scenarios” discussed below) that replicated the intensity of a crisis situation, but which trainees experienced uninterrupted, without the intervention of moment-to-moment feedback from the training team. This continuum of scenarios provided a progressive format to the training through repeated opportunities to observe, discuss, and rehearse de-escalation strategies in scenarios designed with incremental increases in complexity and intensity. The efficacy of scaffolded “rehearsal learning” is supported by recent studies suggesting that the intentional encoding of knowledge through practice-based education can offset the deleterious effects of stress on memory and the retrieval of information (Smith et al. 2016). Moreover, as researchers of critical decision-making and judgment have argued, embodied practice through simulations can expand the repertoire of available patterns of action stored in the memory, allowing skilled intuition in stressful situations to develop (Kahneman and Klein 2009). Rehearsal learning enabled a progressive deepening of tacit knowledge through embodied practice, which contributed toward increased resilience, elevating trainees’ stress tolerance while building confidence in the core competencies of relational policing and de-escalation in situations that might otherwise call for a use of force.

In keeping with Beckley’s contention that “the profession of policing is competency-based” (2004, p. 1), the design of each Forum and Circuit Scenario centred on core competencies in de-escalation tactics, communication and relational policing identified in the multi-perspective assessment tool developed by our team of clinicians, people with lived experience of mental illness, community mental health advocates, researchers, and police trainers. Competency-based learning (CBL) refers to a pedagogical approach that focuses on demonstrable, behavioural outcomes with a particular emphasis on the ability to “apply knowledge in practical real-life situations” (Henri et al. 2017, p. 608). This competency-based approach to the scenario design and assessment served two key functions: 1) it allowed our team of community stakeholders, particularly those who stand to be most impacted by police response, to determine what effective de-escalation looks and feels like; and 2) it ensured that our scenario design was focused on providing a high-fidelity *context* for the application and demonstration of core competencies in dynamic and fluid situations. The authenticity of the scenarios was critical to the realization of CBL-driven outcomes: the opportunity to experience de-escalation competencies “at work” in a crisis situation made the stakes and use-value of the learning manifest, allowing the knowledge—that would otherwise remain notional and theoretical—to be integrated in practice.

### Curriculum Structure

The program begins with a 10-min crisis simulation, undertaken individually by all learners at the outset of training, that was developed for a baseline measurement of skillset to track learner improvement. Learners then move into a series of three 90-min Forum Scenarios allowing learners to engage in the stop-playback method of collective problem-solving in a diversity of high-stakes crisis encounters. Finally, four Circuit Scenarios are staged involving a series of short, high-intensity encounters that each learner undertakes on a “track” in succession, a structure derived from the medical training model of Objective Structured Clinical Examination (OSCE) but familiar to officers who regularly undergo “block training”, which consists of a series of scenarios they move through in succession. This series of intensive scenarios allows for trainer assessment of learner’s abilities to make refined ethical decisions and engage in effective communication, relational approaches, and de-escalation strategies while under stress (Armstrong et al. 2014).

## Conclusion

This conceptual and methodological paper assembled the current empirical landscape of police responses to people in mental health crisis and police training? to outline challenges and advance the argument for the fundamental role of collaborative co-development by diverse stakeholders to bring about innovative specialized training for frontline police officers. Despite increases in the delivery of mental health training programs to law enforcement over the past two decades (Fiske et al. 2020), frontline police training and practices in crisis response continue to underserve people in mental health crisis. There is a well-defined necessity to involve community experts and stakeholders as partners in mental health crisis training design and production to advance training content that serves the needs of citizens as well as police (Coleman and Cotton 2014).

Partnership building and collaborative focus groups among SMEs from across multiple arenas of practice brought to fruition new conceptual insights and a path-breaking specialized training curriculum. Group work on officer’s core competencies illuminated a *relational policing approach* with elements rooted in genuine, humanized, and empathetic responses to persons in crisis. The approach necessitates slowing down and taking time to build trust and an understanding of the person’s needs through active listening and working together to resolve the crisis. Relational approaches are procedurally just and person-centered where the PMHC is viewed as an expert in their own mental health and involved in the decision-making. Such an approach insists that officers are knowledgeable about basic mental illnesses, are trauma-informed, and are self-reflective about potential biases and stereotypes they hold regarding mental illness, which are compounded when the person in mental health crisis is racialized. Officers can build relationships with equity-deserving communities by engaging in culturally safe approaches that encourage self-reflection about the power differentials at play and how their presence shapes the encounter.

A further contribution of this paper details the process behind the co-production of an innovative, theoretically-driven, problem-based scenario paradigm for frontline officers focusing on de-escalation and relational policing in response to mental health crisis. Collaborative procedures resulted in a learning paradigm that brings officers through a spectrum of diverse crisis scenarios using two types of scenarios. The first, Forum Scenarios, are participant-observer simulations informed by Forum Theatre methods. This embodied, experiential learning method employs a stop, playback mode of learning with moment-to-moment SME feedback for repeated opportunities to exercise alternative crisis management and de-escalation techniques in scenarios that might otherwise result in the use of force. The second type of scenario, Circuit Scenarios, offers a series of short, high-intensity evaluation simulations that challenge officers' capacity to use relational strategies, communicate effectively, and make sound decisions. Future research directions include current work to test the efficacy of this training model.

In conclusion, police officers continue to play a pivotal role as first responders in the current context of mental health crisis situations. How officers are trained to approach and resolve mental health crisis calls has profound and far-reaching impacts for those in crisis and their loved ones. To the extent that police officers serve as gatekeepers to both the criminal justice and mental health systems (Canada et al. 2012; Livingston 2016), the continued advancement of specialized response initiatives is essential. Training programs that are coproduced by community experts and stakeholders and prioritize de-escalation and relational policing approaches hold promise as a path forward.
